# L-fucose reduces gut inflammation due to T-regulatory response in Muc2 null mice

**DOI:** 10.1371/journal.pone.0278714

**Published:** 2022-12-30

**Authors:** Natalia A. Feofanova, Victoria D. Bets, Mariya A. Borisova, Ekaterina A. Litvinova

**Affiliations:** 1 Research Institute of Fundamental and Clinical Immunology, Novosibirsk, Russia; 2 Research Institute of Neurosciences and Medicine, Novosibirsk, Russia; 3 Faculty of Physical Engineering, Novosibirsk State Technical University, Novosibirsk, Russia; 4 Federal Research Center Institute of Cytology and Genetics, Siberian Branch of the Russian Academy of Sciences, Novosibirsk, Russia; University of Illinois at Chicago, UNITED STATES

## Abstract

Fucose, the terminal glycan of the intestinal glycoprotein Mucin2, was shown to have an anti-inflammatory effect in mouse colitis models and modulate immune response due to macrophage polarization changes. In this study we evaluated the effect of 0.05% L-fucose supplementation of drinking water on immune parameters in the intestine of homozygous mutant *Muc2*^*−/−*^, compared to *Muc2*^*+/+*^ mice. To get into innate and adaptive immunity mechanisms of gut inflammation, we tested *Prkdc*^*SCID*^*Muc2*^*−/−*^ strain, *Muc2* knockout on SCID background, that is characterized by lack of lymphocytes, in comparison with *Prkdc*^*SCID*^ mice. We evaluated intestinal cytokine profiling, macrophage and eosinophil infiltration, and expression of *Nos2* and *Arg1* markers of macrophage activation in all strains. Markers of Th1, Treg and Th17 cells (*Tbx21*, *Foxp3*, and *Rorc* expression) were evaluated in *Muc2*^*−/−*^ and *Muc2*^*+/+*^ mice. Both *Muc2*^*−/−*^ and *Prkdc*^*SCID*^*Muc2*^*−/−*^ mice demonstrated increased numbers of macrophages, eosinophils, elevated levels of TNFa, GM-CSF, and IL-10 cytokines. In *Muc2*^*−/−*^ mice we observed a wide range of pro-inflammatory cytokines elevated, such as IFN-gamma, IL-1b, IL-12p70, IL-6, M-CSF, G-CSF, IL-17, MCP-1, RANTES, MIP1b, MIP2. *Muc2*^*−/−*^ mice demonstrated increase of *Nos2*, *Tbx21* and *Foxp3* genes mRNA, while in *Prkdc*^*SCID*^*Muc2*^*−/−*^ mice *Arg1* expression was increased. We found that in *Muc2*^*−/−*^ mice L-fucose reduced macrophage infiltration and IL-1a, TNFa, IFNgamma, IL-6, MCP-1, RANTES, MIP1b levels, decreased *Nos2* expression, and induced the expression of Treg marker *Foxp3* gene. On the contrary, in *Prkdc*^*SCID*^*Muc2*^*−/−*^ mice L-fucose had no effect on macrophage and eosinophil numbers, but increased TNFa, GM-CSF, IL-12p70, IL-6, IL-15, IL-10, MCP1, G-CSF, IL-3 levels and *Nos2* gene expression, and decreased *Arg1* gene expression. We demonstrated that anti-inflammatory effect of L-fucose observed in *Muc2*^*−/−*^ mice is not reproduced in *Prkdc*^*SCID*^*Muc2*^*−/−*^, which lack lymphocytes. We conclude that activation of Treg cells is a key event that leads to resolution of inflammation upon L-fucose supplementation in *Muc2*^*−/−*^ mice.

## Introduction

Inflammatory bowel disease (IBD) is a group of conditions that are characterized by chronic inflammation of the gastrointestinal tract. Individuals with IBD have an increased risk of developing colorectal cancer, lymphoma, and biliary cancer [1, 2]. The pathogenesis of IBD is not well understood, but it is believed that the uncontrolled immune response of genetically predisposed individuals to environmental factors and intestinal microorganisms is the cause [3].

Mucin 2 knockout mice strain (*Muc2*^*−/−*^) is one of the genetic models for studying intestinal inflammation [4]. Colitis in *Muc2*^*−/−*^ mice has similar features to ulcerative colitis in humans [5]. Mucin 2 is a gel-forming mucin on the apical surface of the intestinal epithelial cells that acts as a physical barrier and protects cells from the contact with bacteria [6]. In colon specimens from ulcerative colitis patients *Muc2* expression is significantly decreased compared to that from healthy individuals [7]. In mice, homozygous mutation of *Muc2* gene results in the loss of the intestinal barrier integrity, spontaneous colitis over 7 weeks, and development of adenocarcinomas in 6–12 months [8, 9].

Standard non-invasive approaches for IBD treatment include immune-suppressants, which cause significant side effects, show limited effectiveness and long-term remission [10]. The dietary adjustment is now assumed as one of the alternative approaches for ameliorating ulcerative colitis, e.g. with fucoidans, that demonstrate prominent immunomodulatory effects. Fucoidan polysaccharides found in the cell walls of brown seaweeds and in some marine invertebrates are composed of significant amount of fucose and sulfate groups, and lesser amount of arabinose, galactose, glucose, glucuronic acid, mannose, rhamnose, and xylose [11]. Fucoidans are shown to have anti-inflammatory properties in mouse colitis models, however their immunomodulatory effects are strongly dependent on source and chemical composition: Matsumoto et al. [12] demonstrated that fucoidans derived from *Cladosiphon okamuranus Tokida* had anti-inflammatory effect characterized by decreased synthesis of IFN-γ and IL-6 and increased levels of IL-10 and TGF-β in lamina propria of the colon, while *Fucus vesiculosus* fucoidan demonstrated no such effect. The difference in the chemical composition and monosaccharide ratio of fucoidans is critical for the immunomodulatory effects: *Turbinaria decurrens* polysaccharide with ratio Fuc:Gal:Xyl:Man:Rha 6.0:1.3:1.1:1.0:0.6 reduces COX-2 and IL-1β genes expression [13], *Undaria pinnatifida* fucoidan, which contains fucose and galactose in ratio 1.1:1.0, is reported to increase level of IFN-gamma, without influence on other pro-inflammatory cytokines [14]. *Ascophyllum nodosum* fucoidan, with Fuc:Xyl:Gal ratio relatively 10:1:1, exerts pro-inflammatory effects increasing the production of IL-6, IL-8, and TNFa in neutrophils [15].

The main component of fucoidans, monosaccharide L-fucose, also has been shown to exert immunomodulatory and anti-inflammatory effects in DSS-induced acute colitis model [16]. Being the terminal glycan chain component of the glycoprotein Mucin2, orally administered L-fucose could play an important role in modulation of intestine microenvironment in Mucin2-deficient mice. L-fucose has been shown to inhibit macrophage M1 polarization [16, 17]. The resolution of intestinal inflammation and mucosal healing is under the control of local macrophages which are divided into two categories: inflammatory recruited macrophages differentiated from monocytes which migrate to the cite of inflammation (M1 type) and resident macrophages with tolerant phenotype which produce anti-inflammatory cytokines such as IL-10 and TGF-β (M2 type). The shift to M2 appears indispensable to control inflammation and limit tissue injury [18].

The aim of our study was to evaluate the effect of L-fucose oral supplementation on immune parameters in the intestine of *Muc2*^*−/−*^ and control *Muc2*^*+/+*^ mice and estimate its therapeutic potential for IBD treatment. It was of particular interest to get into innate and adaptive immunity mechanisms of gut inflammation and access the effect of L-fucose on intestinal macrophage function in *Prkdc*^*SCID*^*Muc2*^*−/−*^ mice, lacking T and B cells.

## Results

We assessed the effect of 0.05% L-fucose supplementation in drinking water on histological parameters of distal colon, immune cell numbers, cytokine and chemokine levels, and gene expression in *Muc2*^*−/−*^ mice. *Muc2*^*+/+*^ mice were used as a control group. To get into innate and adaptive immunity mechanisms of gut inflammation, we tested *Prkdc*^*SCID*^*Muc2*^*−/−*^ strain, using *Prkdc*^*SCID*^ mice as a control.

**Fig 1:**
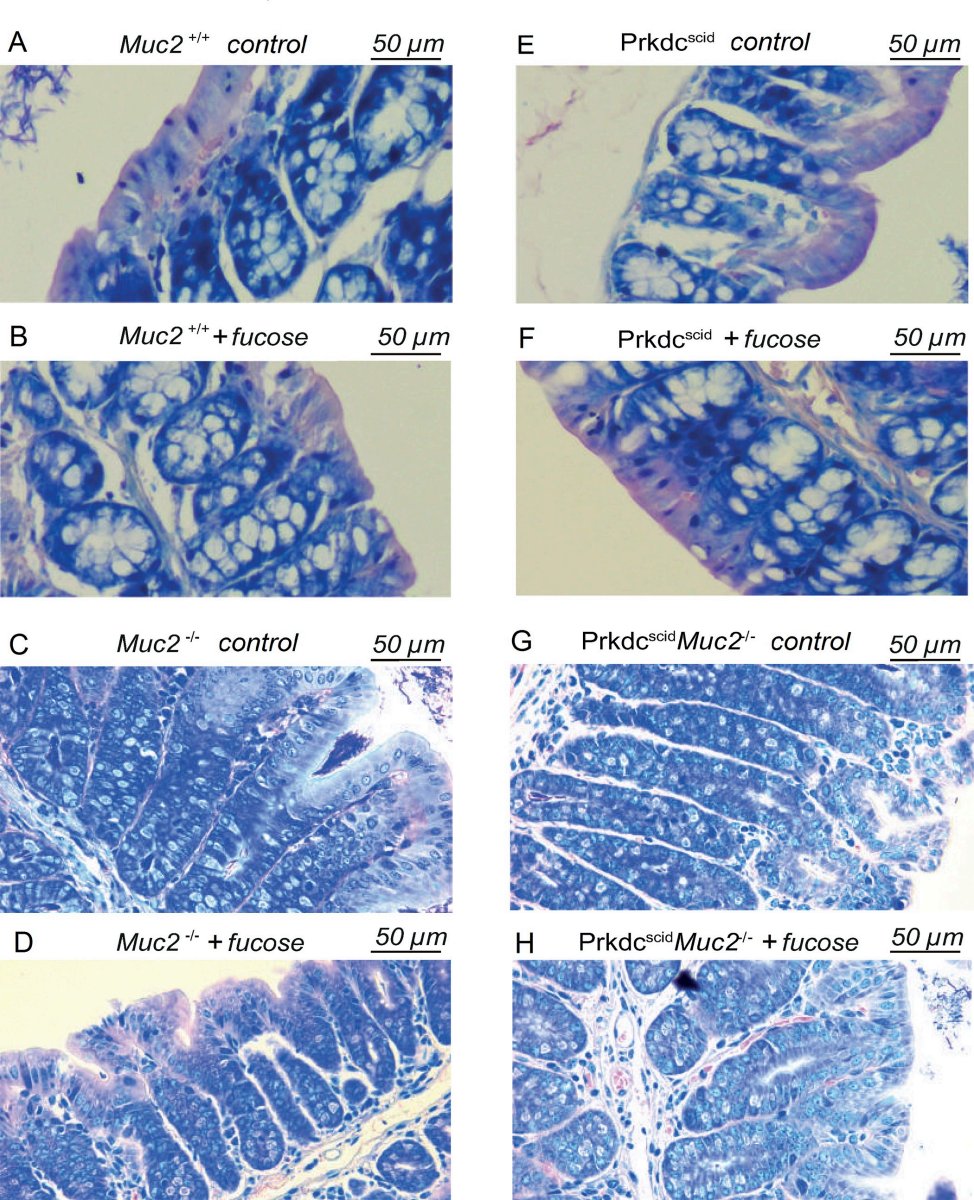
PAS-stained histological colon sections. Control *Muc2*^*+/+*^ mice (A), L-fucose-treated *Muc2*^*+/+*^ mice (B), control *Muc2*^*−/−*^ mice (C), L-fucose-treated *Muc2*^*−/−*^ mice (D), control *Prkdc*^*SCID*^ mice (E), L-fucose-treated *Prkdc*^*SCID*^ mice (F), control *Prkdc*^*SCID*^*Muc2*^*−/−*^ mice (G), L-fucose-treated *Prkdc*^*SCID*^*Muc2*^*−/−*^ mice (H).

**Fig 2:**
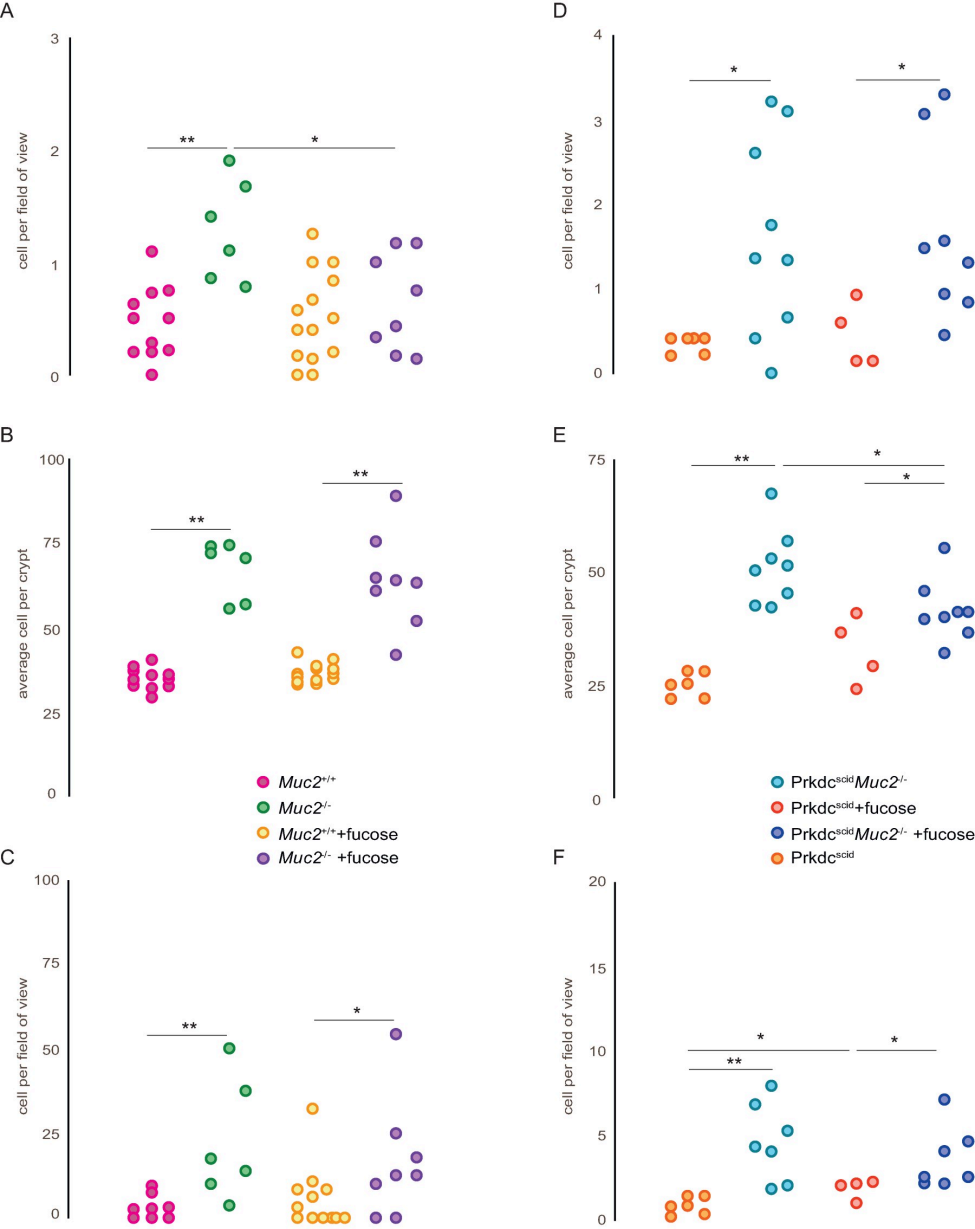
Histological markers of inflammation in the distal colon. (* = p < 0.05, ** = p < 0.01, Mann-Whitney u-test) (A) Number of macrophages per field of view in *Muc2*^*−/−*^ and *Muc2*^*+/+*^ mice with and without L-fucose treatment, (B) Number of cells per crypt in *Muc2*^*−/−*^ and *Muc2*^*+/+*^ mice with and without L-fucose treatment (C) Number of eosinophils in *Muc2*^*−/−*^ and *Muc2*^*+/+*^ with and without L-fucose treatment (D) Number of macrophages per field of view in *Prkdc*^*SCID*^*Muc2*^*−/−*^ and *Prkdc*^*SCID*^ mice with and without L-fucose treatment (E) Number of cells per crypt in *Prkdc*^*SCID*^*Muc2*^*−/−*^ and *Prkdc*^*SCID*^ mice with and without L-fucose treatment (F) Number of eosinophils in *Prkdc*^*SCID*^*Muc2*^*−/−*^ and *Prkdc*^*SCID*^ mice with and without L-fucose treatment.

We observed significant difference in the colon morphology between *Muc2*^*−/−*^ and *Muc2*^*+/+*^ mice, that was manifested as an increase in the number of cells per crypt, or crypt hyperplasia (p = 0.001) (Figs 1A, 1C and 2B). *Muc2*^*−/−*^ mice demonstrated significant macrophage (p = 0.003) and eosinophil (p = 0.001) infiltration in the distal colon compared to *Muc2*^*+/+*^ mice (Fig 2A and 2C). L-fucose treatment of *Muc2*^*−/−*^ mice resulted in a decrease of the macrophages number (p = 0.031) to the levels comparable to those observed in gut of *Muc2*^*+/+*^ mice (Fig 2A), however, there was no statistically significant effect of L-fucose on the number of eosinophils and crypts hyperplasia (Fig 2B and 2C). *Prkdc*^*SCID*^*Muc2*^*−/−*^ mice demonstrated histological changes, similar to those in *Muc2*^*−/−*^ mice: macrophages (p = 0.034), eosinophils (p = 0.002) and cells per crypt numbers (p = 0.002) were increased compared to *Prkdc*^*SCID*^ mice (Figs 1E, 1G, 2D–2F). L-fucose treatment of *Prkdc*^*SCID*^*Muc2*^*−/−*^ mice had no effect on the number of macrophages and eosinophils, but caused a moderate decrease of crypts hyperplasia (p = 0.016) (Figs 1G, 1H and 2E). We also observed slight but statistically significant increase of eosinophils number in *Prkdc*^*SCID*^ mice under L-fucose treatment (p = 0.031) (Fig 2F).

**Fig 3:**
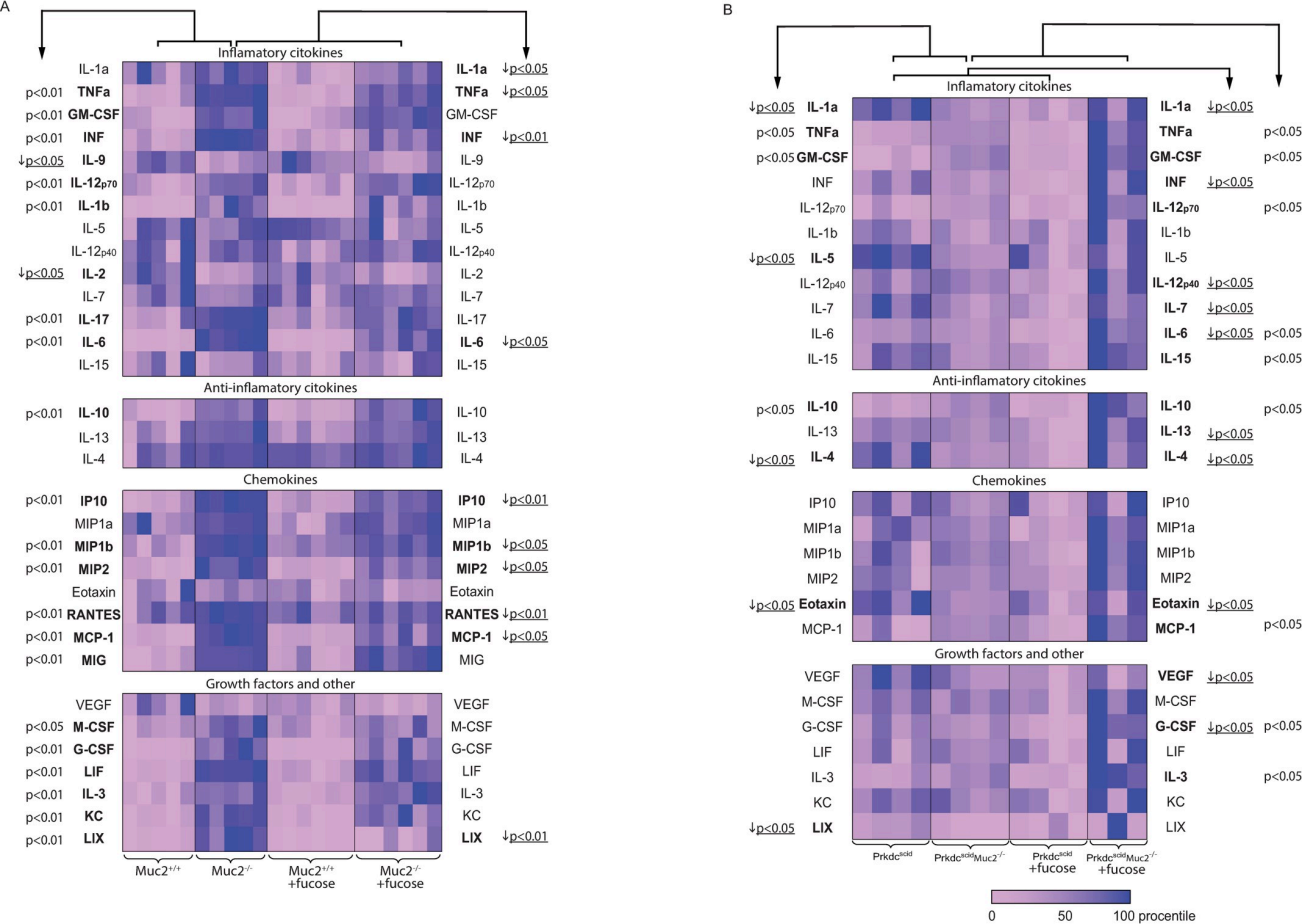
Heat map of cytokine levels in the distal colon. (* = p < 0.05, ** = p < 0.01, Mann-Whitney u-test). (A) *Muc2*^*−/−*^ and *Muc2*^*+/+*^ mice with and without L-fucose treatment. (B) *Prkdc*^*SCID*^*Muc2*^*−/−*^ and *Prkdc*^*SCID*^ mice with and without L-fucose treatment.

We next evaluated immunologic milieu in the gut of *Muc2*^*−/−*^ mice with the analysis of cytokines, chemokines and growth factors panel (Fig 3). We observed strong up-regulation of the following pro-inflammatory cytokines levels in M*uc2*^*−/−*^ compared to *Muc2*^*+/+*^ mice: TNFa, GM-CSF, G-CSF, IFNgamma, IL-1b, IL-12p70, IL-6, G-CSF, IL-17, MCP-1, RANTES, MIP1b, MIP2, MIG, IP10, LIF, IL3, KC, LIX (p = 0.009), and M-CSF (p = 0.016). The level of anti-inflammatory cytokine IL-10 was increased, while IL-2 (p = 0.028) and IL-9 (p = 0.047) levels were decreased. L-fucose in *Muc2*^*−/−*^ mice induced strong down-regulation of the following pro-inflammatory cytokines levels: IL-1a, MIP1b, MCP-1 (p = 0.028), TNFa (p = 0.045), IFNgamma (p = 0.006), IL-6, MIP2 (p = 0.018), RANTES, LIX, IP10 (p = 0.011) (Fig 3A). In *Prkdc*^*SCID*^*Muc2*^*−/−*^ mice we observed moderate inflammation with only TNFa, GM-CSF, and IL-10 (p = 0.021) levels increased, compared to *Prkdc*^*SCID*^ mice. Levels of IL-1a, IL-5, IL-4, LIX (p = 0.021), and Eotaxin (p = 0.043) in *Prkdc*^*SCID*^*Muc2*^*−/−*^ were even lower than in *Prkdc*^*SCID*^ mice. We observed significant effect of L-fucose on *Prkdc*^*SCID*^ mice cytokine levels: decrease of IL-1a, IFNgamma, IL-4, IL-6, IL-7, IL-13, VEGF, G-CSF (p = 0.021), IL-12p40, Eotaxin (p = 0.043). In *Prkdc*^*SCID*^*Muc2*^*−/−*^ mice L-fucose increased levels of TNFa, GM-CSF, IL-12p70, IL-6, IL-15, IL-10, MCP1, G-CSF, IL-3 (p = 0.034) (Fig 3B).

To characterize immune state of gut macrophages and T-cells we assessed the expression of some major genes involved in macrophage and T-cells activation. We measured *Nos2* expression as a marker of pro-inflammatory M1, and *Arg1* expression as a marker of M2 subpopulation of macrophages. In *Muc2*^*−/−*^ mice we also measured the expression of main T-cell markers, *Tbx21* for Th1, and *Foxp3* for T-reg and *Rorc* for Th17 subpopulation.

**Fig 4:**
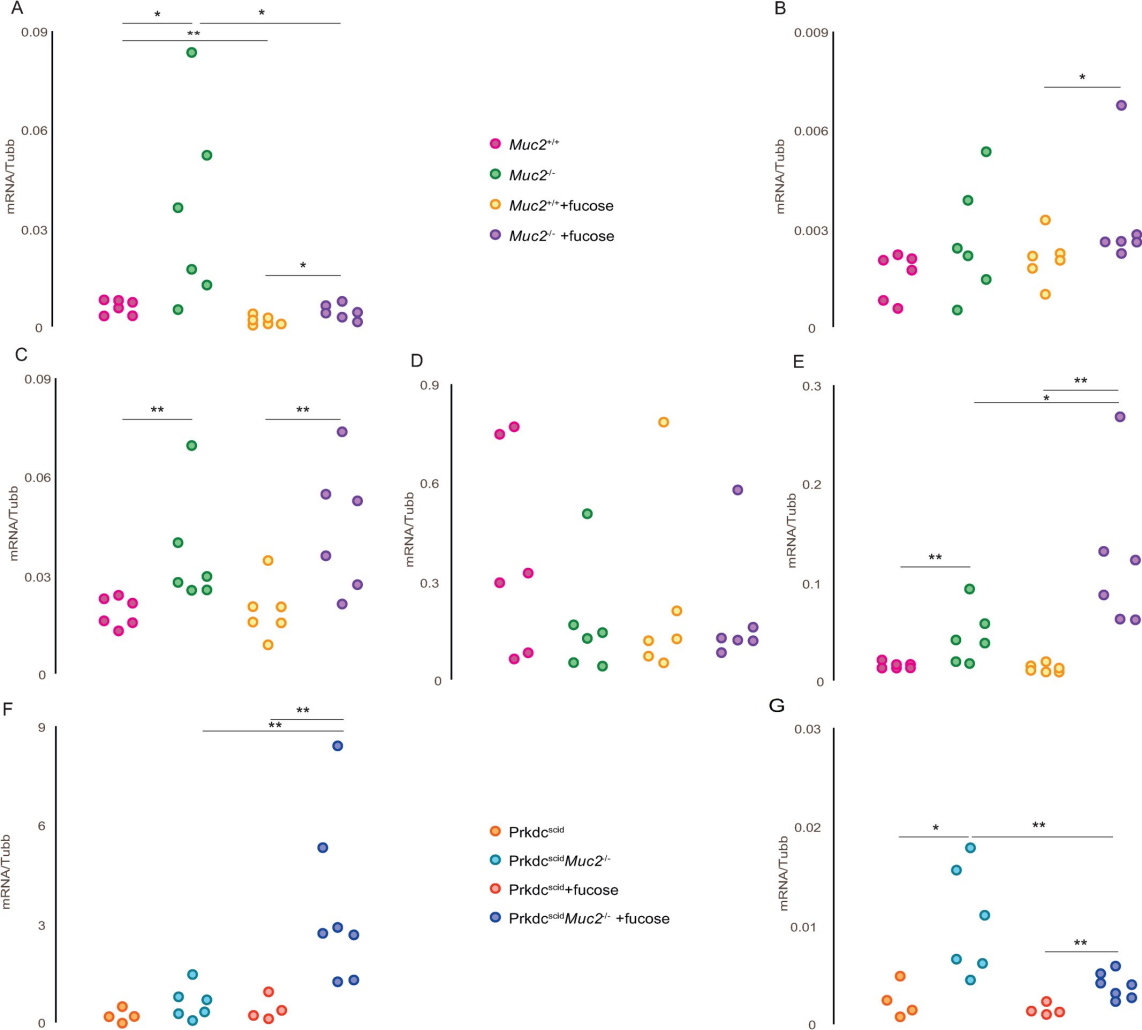
Expression of macrophage and T-cell activation marker genes in the distal colon. (* = p < 0.05, ** = p < 0.01, Mann-Whitney u-test). (A) *Nos2* gene expression in *Muc2*^*−/−*^ and *Muc2*^*+/+*^ mice with and without L-fucose treatment. (B) *Arg1* gene expression in *Muc2*^*−/−*^ and *Muc2*^*+/+*^ mice with and without L-fucose treatment. (C) *Tbx21* gene expression in *Muc2*^*−/−*^ and *Muc2*^*+/+*^ mice with and without L-fucose treatment. (D) *Rorc* gene expression in *Muc2*^*−/−*^ and *Muc2*^*+/+*^ mice with and without L-fucose treatment. (E) *Foxp3* gene expression in *Muc2*^*−/−*^ and *Muc2*^*+/+*^ mice with and without L-fucose treatment. (F) *Nos2* gene expression in *Prkdc*^*SCID*^*Muc2*^*−/−*^ and *Prkdc*^*SCID*^ mice with and without L-fucose treatment. (G) *Arg1* gene expression in *Prkdc*^*SCID*^*Muc2*^*−/−*^ and *Prkdc*^*SCID*^ mice with and without L-fucose treatment.

The evaluation of *Nos2* and *Arg1* genes expression analysis in the distal colon demonstrated elevated level of *Nos2* gene mRNA in *Muc2*^*−/−*^ compared to *Muc2*^*+/+*^ mice (p = 0.025) (Fig 4A). L-fucose significantly decreased *Nos2* gene expression both in *Muc2*^*−/−*^ (p = 0.016) and *Muc2*^*+/+*^ (p = 0.004) groups (Fig 4A). We observed no statistically significant effect of L-fucose on *Arg1* gene expression. (Fig 4B). L-fucose increased *Nos2* gene expression in *Prkdc*^*SCID*^*Muc2*^*−/−*^ mice (p = 0.008) (Fig 4F). *Arg1* gene expression was significantly higher in *Prkdc*^*SCID*^*Muc2*^*−/−*^ compared to *Prkdc*^*SCID*^ mice (p = 0.019) and L-fucose caused a decrease of *Arg1* gene expression in *Prkdc*^*SCID*^*Muc2*^*−/−*^ (p = 0.008) (Fig 4F and 4G). Significant increase of *Tbx21* (p = 0.004) and *Foxp3* (p = 0.01) genes expression typical for Th1 and Treg cells, correspondingly, was observed in distal colon of *Muc2*^*−/−*^ mice, compared to *Muc2*^*+/+*^ mice (Fig 4C and 4E). The expression of Th17 cell *Rorc* gene did not differ between *Muc2*^*−/−*^ and *Muc2*^*+/+*^ strains. (Fig 4D). We observed no statistically significant effect of L-fucose on *Tbx21* and *Rorc* gene expression neither in *Muc2*^*−/−*^ nor in *Muc2*^*+/+*^ mice (Fig 4C, 4D). L-fucose significantly increased *Foxp3* expression in *Muc2*^*−/−*^ mice (p = 0.016), but not in *Muc2*^*+/+*^ mice (Fig 4E).

Hereby, L-fucose oral administration in *Muc2*^*−/−*^ mice resulted in profound anti-inflammatory effect causing reduction of macrophage infiltration and IL1a, TNFa, IFNgamma, IL6, MCP-1, RANTES, MIP1b levels, decrease of *Nos2* gene and increase of *Foxp3* gene expression. This effect was not reproduced in *Prkdc*^*SCID*^*Muc2*^*−/−*^ mice that lack lymphocytes, suggesting that enhancement of Treg cells activity is indispensable for the L-fucose effect. The decrease of inflammatory markers due to L-fucose treatment in *Muc2*^*+/+*^ mice seems to be secondary to Treg cells activation.

## Discussion

The damage of intestinal epithelial cell barrier stimulates macrophages, leading to pro-inflammatory cytokine production and reactive oxygen and nitrogen intermediates release. As a result, intestinal epithelial cells undergo injury followed by cell death which promotes the occurrence and development of IBD. Disturbed immunological homeostasis, oversecreting of cytokines and chronic inflammation are typical features of IBD [3]. Here, in *Muc2*^*−/−*^ mice model we demonstrated the presence of histological inflammatory markers of IBD such as crypts hyperplasia, increased colon macrophage and eosinophil numbers, and strong up-regulation of the pro-inflammatory cytokines.

Polarization of macrophages to M1 or M2 state is *in vitro* phenomenon, while *in vivo* stimulation with multiple signals produces the mixture of macrophages being at different activation stages. Some studies have shown that macrophages become a continuum of activation states when they are stimulated by certain cytokines such as TNF-α, LPS, TGF-β, IL-10, IL-13 [19–22]. In our study we also showed such continuum of macrophage activation states in the intestine with prevalence of pro-inflammatory markers: enhanced *Nos2* gene expression and elevated pro-inflammatory cytokines (TNFa, GM-CSF, M-CSF, G-CSF, IFNgamma, IL-1b, IL-12p70, IL-6, IL-17, G-CSF) as well as chemokines of monocytes, NK cells, and T cells (MCP-1, RANTES, MIP1b, MIP2, MIG, IP10, LIF, IL3, KC, LIX). At the same time, some pro-inflammatory cytokines, such as IL-2 and IL-9, were decreased, compared to control levels in *Muc2*^*+/+*^ mice, while anti-inflammatory IL-10 level was increased. IL-9 is associated with Th2 response, inhibition of cellular proliferation and impairment of intestinal epithelial layer repair [23], its decreased expression level is consistent with enhanced proliferation and crypts hyperplasia in *Muc2*^*−/−*^ mice. High level of IL-10 is in good agreement with enhanced *Foxp3* expression in *Muc2*^*−/−*^ mice colon, as IL-10 produced by macrophages has the stimulatory effect of Foxp3^+^ Treg cells numbers [24]. We did not observe the elevation of Th2 cytokines such as IL-4, IL-5, IL-13, but *Foxp3* and *Tbx21* genes expressions were increased in *Muc2*^*−/−*^ mice.

In human, IBD encompasses Crohn’s disease and ulcerative colitis. Patients with Crohn’s disease have increased amount of Th1-related cytokines, while patients with ulcerative colitis exhibit a more pronounced Th2-related immune response [25, 26]. *Muc2*^*−/−*^ mice are assumed as a model for human ulcerative colitis: *Muc2*^*−/−*^ mice and ulcerative colitis patients have elevated colon neutrophils, T-cells and macrophages while a reduced frequency of CD103^+^ dendritic cells [5]. Here, we report more detailed information on cytokine profile in *Muc2*^*−/−*^ mice model that could be useful for translation from animal models results to patients in terms of immune status.

L-fucose has been shown to inhibit macrophage M1 polarization and ameliorate DSS-induced acute colitis [16]. However, in our previous study, L-fucose improved inflammation neither in acute, nor in chronic DSS-induced colitis [27]. In our previous work we showed the importance of the immune context for the effect of L-fucose on macrophage polarization and pregnancy outcome: two prototypical mouse strains with Th1- and Th2-type of immune response demonstrated the opposite effect of L-fucose treatment [17]. These data suggest that L-fucose effects on inflammation are highly dependent on the current immune state and microenvironment which in *in vivo* context includes immune-system-microflora interactions. In our study we excluded the factors of genetic background and microflora perturbations and focused on the role of immune cell regulation comparing the effect of L-fucose on *Muc2*^*−/−*^ and *Prkdc*^*SCID*^*Muc2*^*−/−*^ mice which lack B and T-cells with the control *Muc2*^*+/+*^ and *Prkdc*^*SCID*^ strains.

L-fucose supplementation in *Muc2*^*−/−*^ mice significantly decreased macrophage infiltration, *Nos2* gene expression in colon, induced strong down-regulation of pro-inflammatory cytokines levels: IL-1a, TNFa, IFNgamma, IL-6, MCP-1, RANTES, MIP1b, MIP2. L-fucose significantly increased *Foxp3* gene expression in *Muc2*^*−/−*^ mice, but not in *Muc2*^*+/+*^ mice, suggesting that L-fucose induces Treg cells activation indirectly, in context of the inflammatory microenvironment. In *Muc2*^*−/−*^ mice, characterized by markers of M1 macrophages and acute inflammation, together with high expression levels of Th1 and Treg cells, L-fucose oral administration further enhanced activation of Treg cells, which became a key event that drives immune equilibrium from the chronic inflammatory state to its resolution. In *Prkdc*^*SCID*^*Muc2*^*−/−*^ mice, characterized by only macrophage-dependent moderate inflammatory profile, increased TNFa, GM-CSF, IL-10 levels and *Arg1* gene expression, L-fucose had no anti-inflammatory effect. We assume that in *Muc2*^*−/−*^ mice Treg cells are the main effectors of the anti-inflammatory effect of L-fucose. It is of interest that in *Prkdc*^*SCID*^*Muc2*^*−/−*^ mice L-fucose had even moderate pro-inflammatory effect causing increase of *Nos2* gene expression, TNFa, GM-CSF, IL-12p70, IL-6, IL-15, IL-10, MCP1, G-CSF, IL-3 levels, and a decrease of *Arg1* gene expression. The pro-inflammatory effect of L-fucose in *Prkdc*^*SCID*^*Muc2*^*−/−*^ mice could be due to shifts in microflora composition. It has been shown that in patients with Crohn’s disease with adherent-invasive *E*. *coli* enriched in the intestinal microbiota, propionate production by adherent-invasive *E*. *coli*. depends on L-fucose availability. Propionate synergizes with lipopolysaccharide to induce IL-1β production by macrophages, and disrupting of fucose availability limits microbial propionate and intestinal inflammation [28].

Microflora plays an important role in intestinal homeostasis. Certain bacterial species can influence function of regulatory T-cells. Polysaccharide A of *B*. *fragilis* mediates the conversion of CD4^+^ T cells into Foxp3^+^ Treg cells and is capable of reversing experimental colitis in mice [29]. *Clostridium spp*. and *Helicobacter spp*. can induce accumulation of Treg cells in the gut lamina propria [30, 31]. *Ruminococcaceae*, *Eubacterium*, *Clostridia*, and *Firmicutes* are identified as the main producers of butyrate [32], which can exert anti-inflammatory effect by suppressing the activation of NF-κB [33], inhibiting IFN-γ signaling [34], or directrly promoting differentiation of Treg cells [35]. Thereby, microbiota can influence Treg cells generation in the gut via numerous ways, including metabolic products, such as propionate and butyrate, modulation of cytokine signaling, and even via direct contact with TLRs on T cells [36]. Besides involvement in microbial metabolism, L-fucose can change bacterial immunogenic properties being the component of bacterial cell wall polysaccharides. Fucosylated polysaccharides of *Fasciola hepatica* recognized by dendritic cell receptor DC-SIGN can induce tolerogenic response associated with IL-10 production [37].

Modulation of microbiota by L-fucose is demonstrated in the model of DSS-induced colitis and seems to be tightly associated with its protective effects, as in antibiotic-treated mice L-fucose does not ameliorate colitis [38]. Supplementation of drinking water with L-fucose in *Muc2*^*−/−*^ mice undergoing antibiotics treatment also does not reduce the inflammatory response, but partially restores the level of *Bacteroides spp*., and improves some biochemical parameters [39]. Direct effect of L-fucose on macrophages was also demonstrated, L-fucose inhibited macrophage activation and inflammatory cytokine production *in vitro* [16]. L-fucose can exert its anti-inflammatory action in different ways including direct effect on macrophage and dendrite cells and modulation of antigen-presentation process via microflora alteration. We showed that *in vivo* T-cells are indispensable for this process, however further research is required for understanding of the molecular and cellular mechanisms of L-fucose-induced decrease of intestinal inflammation in *Muc2*^*−/−*^ mice.

## Materials and methods

### Animal housing

The study was performed in the Center for Genetic Resources of Laboratory Animals at the Federal Research Center Institute of Cytology and Genetics of The Siberian Branch of the Russian Academy of Sciences (ICG SB RAS). All procedures were conducted under Russian legislation according to Good Laboratory Practice standards (directive # 267 from 19 June 2003 of the Ministry of Health of the Russian Federation), inter-institutional bioethical committee guidelines, and the European Convention for the protection of vertebrate animals used for experimental and other scientific purposes; all procedures were approved by the bioethical committee, protocol #18.6 (14 October 2013). All animals used had specific pathogen free (SPF) status, which was tested quarterly according to FELASA recommendations [40].

*Muc2*^*−/−*^ mice were obtained from the Federal Research Centre “Fundamentals of Biotechnology” of the Russian Academy of Sciences (Moscow, Russia). *Muc2*^*+/−*^ mice were generated by crossing *Muc2*^*−/−*^ on C57BL/6 genetic background males to C57BL/6 females. *Muc2*^*+/+*^ and *Muc2*^*−/−*^ mice generated by crossing of *Muc2*^*+/−*^mice were used in the experiments. In experiments with *Prkdc*^*SCID*^ and *Prkdc*^*SCID*^*Muc2*^*−/−*^, offspring from outbred mice was used. *Prkdc*^*SCID*^*Muc2*^*+/−*^ mice were generated by crossing females of the outbred strain *SHO-Prkdc*^*SCID*^Hr^*hr*^ (Charles River, France) with inbred *Muc2*^*−/−*^ males. *Prkdc*^*SCID*^*Muc2*^*+/−*^ mice were generated by crossing of *Prkdc*^*Wt/SCID*^*Muc2*^*+/−*^ heterozygotes. *Prkdc*^*SCID*^*Muc2*^*+/−*^ we used to generate mice of *Prkdc*^*SCID*^ and *Prkdc*^*SCID*^*Muc2*^*−/−*^ genotypes.

All animals were kept in groups of three to six same-sex siblings in individually ventilated cages, Optimice (AnimalCare Systems, Centennial, USA). All animals were housed under a 14h/10h light/dark photoperiod at 20–22°C temperature, 30–60% humidity, and 10 air changes per hour; food (ssniff R/M-H autoclavable V1534-3, Spezialdiaeten GmbH, Soest, Germany) and sterile deionized water were provided *ad libitum*. All samples were collected between 12:00 and 16:00 (light time period). Colon samples were taken for histological analysis, cytokine multiplex analysis, real-time PCR. Colon samples were placed in 10% neutral formalin for histological analysis or frozen in liquid nitrogen for gene expression measurements.

### Experimental groups

The experiments on *Muc2*^*+/+*^ and *Muc2*^*−/−*^ mice were performed on female mice obtained by crossing of *Muc2*^*+/−*^ mice. Five days after mating, males were placed in the individual cages. In the experimental groups (“+ fucose”), mothers were provided with drinking water supplemented with 0.05% L-fucose (Biosynth Carbosynth, UK) *ad libitum* from the moment of delivery during 4 weeks. After 4 weeks, the offspring was placed in separate cages in same-sex groups, animals genotype was determined by real-time PCR. After weaning from mothers, animals from the experimental group (“+ fucose”) were provided with 0.05% L-fucose in drinking water *ad libitum*. Animals from the control group were provided with drinking water. At the age of 7 weeks, animals were euthanized using craniocervical dislocation, colon samples were taken for further experiments.

The experiments on *Prkdc*^*SCID*^*Muc2*^*−/−*^ and *Prkdc*^*SCID*^ mice were performed on 8 weeks-old female mice, obtained by crossing of *Prkdc*^*SCID*^*Muc2*^*+/−*^ mice. Due to the technical difficulties at the Center for Genetic Resources of the Institute of Cytology and Genetics of the Siberian Branch of the Russian Academy of Sciences, we had no possibility of adding L-fucose to the drinking water of mothers and had to reduce the number of animals per group. Adult 8 week-old mice of the experimental groups (“+ fucose”) were provided with 0.1% L-fucose in drinking water *ad libitum* during 2 weeks. Due to the insufficient number of animals in *Prkdc*^*SCID*^ groups (control and “+ fucose”) 4 mice were taken per group. In *Muc2*^*-/-*^ *Prkdc*^*SCID*^ groups, there were 6 females per control and 7 per “+ fucose” group. Female mice of the experimental group (+ fucose) from 8 to 10 weeks-old were provided with 0.1% L-fucose in drinking water *ad libitum*, the concentration was corrected according to the shorter duration of the experiment (2 instead of 4 weeks) and body weight of 8 weeks-old females. At the age of 10 weeks, animals were euthanized using craniocervical dislocation and colon samples were taken for further experiments.

### Histological analysis

Colons were fixed in 10% neutral buffered formalin and embedded in paraffin. Paraffin sections (4 μm) were stained with Periodic acid-Schiff (PAS) stain (BioVitrum, Saint Petersburg, Russia) to examine general morphology and to detect goblet cell secretion. Azur-II-eosin stain was used to detect inflammatory cell infiltration. The sections were examined in a blinded manner. Images were taken with an AxioImager.M2 microscope with N-Achroplan 5×/0.15 and 10×/0.25 objectives using an Axiocam 305 color camera (Zeiss, Germany). The average number of epithelial cells per crypt was counted in PAS-stained sections (15 randomly selected crypts). Hyperplasia was defined as the percentage of cells per crypt above the mean number of those counted in the control sections. Macrophages and eosinophils were counted in azur-II-eosin stained sections in 15 fields of view at × 1000 magnification using Plan-Neofluar 100×/1.30 objective to evaluate inflammation severity.

### Real-time PCR

To measure the levels of genes expression, we purified total RNA from colonic samples using TRIzol reagent (Invitrogen, Waltham, MA, USA) according to the manufacturer’s recommendations. RNA concentration was measured with a NanoDrop 2000 spectrophotometer (ThermoScientific, Waltham, MA, USA). 1 μg of RNA was used in reverse transcription reaction, cDNA synthesis was performed using M-MuLV reverse transcriptase (SibEnzyme, Novosibirsk, Russia) according to the manufacturer’s recommendations. A mix of random hexa-deoxyribonucleotide and Oligo-dT primers were used for reverse transcription, after the completion of the reaction its volume of 20 μL was diluted to 100 μL with deionized water. Real-time PCR reaction was prepared using a BioMaster HS-qPCR SYBR Blue (2x) (BioLabMix, Novosibirsk, Russia), 5 μL of cDNA, and 250 nM specific primers. Amplification and detection were performed using a CFX96 Touch™ Real-Time PCR Detection System (BioRad, Hercules, CA, USA). Gene expression was normalized to *Tubb5* (*tubulin*, *beta 5 class I*) mRNA level as ΔCt = 2 ^ (Ct_Tubb5_ mRNA − Ct_gene of interest_ mRNA). Primer sequences used for real-time PCR analysis: betaTub_F TGAAGCCACAGGTGGCAAGTAT, betaTub_R CCAGACTGACCGAAAACGAAGT; Foxp3_F AGAGTTTCTCAAGCACTGCCA Foxp3_R TCCCAGCTTCTCCTTTTCCA; Rorc_F TGGGCTCCAAGAGAAGAGGA, Rorc_R CAGGCTCCGGAGTTTTCCTT; Arg1_F AAGAGCTGGCTGGTGTGGTG, Arg1_R ACACAGGTTGCCCATGCAGA; Nos2_F ATCGACCCGTCCACAGTATGT, Nos2_R CATGATGGACCCCAAGCAAGA, Tbx21_F CCAGGGAACCGCTTATATG, Tbx21_R CGATCATCTGGGTCACATTGT.

### Immunological multiplex assay

To measure cytokine levels, a distal colon sample was homogenized in liquid nitrogen, 100 μl PBS was added, and then the sample was centrifuged at 12,000 rpm for 15 min at 4°C. Cytokine concentration in the supernatant was measured using MILLIPLEX MAP Mouse Cytokine/Chemokine Magnetic Bead Panel (Merck, Germany) according to the manufacturer’s recommendations. Detection was performed using Luminex 200 System (Merck, Germany) with xPONENT 3.1. software. Cytokine concentration was normalized to total protein, which was measured as described by Bradford, and is presented as pg of cytokine per mg of total protein.

### Data analysis and statistics

The data were tested for normality using the Kolmogorov-Smirnov test. Not normally distributed data were processed using Mann–Whitney *U* test. Significance was determined as p ≤ 0.05. The IBM SPSS Statistics 23 software was used for analysis.
